# Quantum oscillations from generic surface Fermi arcs and bulk chiral modes in Weyl semimetals

**DOI:** 10.1038/srep23741

**Published:** 2016-04-01

**Authors:** Yi Zhang, Daniel Bulmash, Pavan Hosur, Andrew C. Potter, Ashvin Vishwanath

**Affiliations:** 1 Department of Physics, Stanford University, Stanford, California 94305, USA; 2 Department of Physics, University of California, Berkeley, California 94720, USA

## Abstract

We re-examine the question of quantum oscillations from surface Fermi arcs and chiral modes in Weyl semimetals. By introducing two tools - semiclassical phase-space quantization and a numerical implementation of a layered construction of Weyl semimetals - we discover several important generalizations to previous conclusions that were implicitly tailored to the special case of identical Fermi arcs on top and bottom surfaces. We show that the phase-space quantization picture fixes an ambiguity in the previously utilized energy-time quantization approach and correctly reproduces the numerically calculated quantum oscillations for generic Weyl semimetals with distinctly curved Fermi arcs on the two surfaces. Based on these methods, we identify a ‘magic’ magnetic-field angle where quantum oscillations become independent of sample thickness, with striking experimental implications. We also analyze the stability of these quantum oscillations to disorder, and show that the high-field oscillations are expected to persist in samples whose thickness parametrically exceeds the quantum mean free path.

Weyl semimetals are three-dimensional quantum materials characterized by a band gap that closes at isolated points, Weyl nodes, in the Brillouin zone. Weyl nodes serve as sources of quantized monopole fluxes of ±2*π* Berry curvature, whose sign defines a chirality *χ* = ±1 for each node, and hence serves as an example of quantum topology in the absence of a band gap^1^,^2^. At a spatial surface, the bulk band topology produces unusual Fermi-arc surface states, whose Fermi “surface” consists of disjoint arc segments that pairwise connect surface projections of opposite chirality Weyl nodes^1^,^2^,^3^,^4^, and have been observed in photoemission experiments^5^,^6^ and band-structure calculations^7^ on crystalline materials. Moreover, in the presence of a magnetic field, 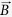, Weyl nodes exhibit chiral Landau level (LL) modes^8^ with field-independent dispersion 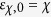
*v*_‖_ *k*_‖_, where *v*_‖_, *k*_‖_ are respectively the velocity and momentum along 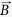.

Recently, it was shown^9^ that an applied magnetic field perpendicular to the surface of a Weyl semimetal drives a novel kind of cyclotron orbit in which electrons slide along a Fermi-arc on the top surface from *χ* = +1 towards *χ* = −1 Weyl nodes, transfers to the bulk chiral LL mode of the *χ* = −1 node on which they propagate to the bottom surface, traverse the bottom Fermi-arc and return to the top surface via the mode with the opposite chirality. Ordinary cyclotron orbits around closed Fermi surfaces of metals are routinely studied via quantum oscillations, periodic-in-1/*B* modulations in the density of states that appear in various thermodynamic and transport properties, and help unveil the detailed structure of the underlying Fermi surface. Ref. 9 showed that the quantized energy levels arising from these mixed surface and bulk cyclotron orbits indeed exhibit periodic quantum oscillations, whose phase exhibits a characteristic dependence on sample thickness that distinguishes them from conventional cyclotron orbits, and hence offering a direct probe of the topological connection between surface Fermi arcs and bulk Weyl bands. Experimental evidence for such quantum oscillations was recently reported in the Dirac semimetal Cd_3_As_2_ 10. In addition, transport experiments were proposed based on the distinctive electronic properties of these cyclotron orbits^11^.

The semiclassical quantization of these cyclotron orbits in ref. 9 was carried out through “energy-time” quantization, by demanding that the product of the energy, *ε*, of the electron and the semiclassical time of the orbit, *t*, equals an integer multiple of 2*π*. Noting that 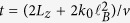, where *k*_0_ is the *k*-space arc length of the Fermi arcs on the top and bottom surfaces, *L*_*z*_ is the sample thickness, *v* is the Fermi velocity, and 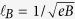 is the magnetic length, the energy-time quantization condition states that the *n*^th^ quantized level crosses the chemical potential, 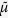 at field *B* = *B*_*n*_:


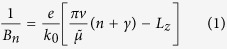


which occur periodically in 1/*B* with period 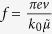 and a thickness dependent phase offset: 
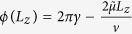
.

However, this approach leaves open a basic question: what is the overall zero of energy for 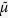
? This issue is experimental pertinent, as it effects the frequency, *f*, of the quantum oscillations. We will show that the nature choice of the energy of the bulk Weyl node corresponds to the special case, implicitly assumed in ref. 9, where the Fermi arcs on the top and bottom surfaces are identical. More generically, however, the Fermi arcs may have different shapes, and the zero of energy need not coincide with the Weyl node energy.

To generalize the results of ref. 9 to include the generic case with arbitrarily curved Fermi arcs, we adopt an alternative phase-space quantization perspective in which the integral of momentum times spatial displacement is equal to 
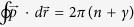
 for integer *n* and a constant quantum offset *γ*. Comparison to the energy-time quantization transparently identifies the zero of energy as where the surface arcs enclose zero *k*-space area using appropriate extrapolation from the chemical potential to lower energy. This method also predicts an additional thickness dependent correction to Eq. 1, which is difficult to obtain from the energy-time quantization perspective.

We also discuss the experimental consequences of our results. First, we identify a special set of ‘magic’ angles of the magnetic field, for which the length-dependence of the phase of the quantum oscillations drops out. We explain how this effect enables a smoking gun signature of quantum oscillations from surface Fermi arcs in recently measured thin-film devices with non-parallel surfaces^10^. Second, we examine the effects of impurities, and find that these quantum oscillations are surprisingly resilient to disorder for sufficiently strong fields. In contrast to conventional quantum oscillations, which are obscured by disorder unless the cyclotron orbit is smaller than the quantum mean free path 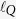, we find that for strong fields, quantum oscillations from surface Fermi arcs and bulk chiral modes can persist in samples whose thickness substantially exceeds 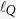.

Finally, we construct a tight-binding model based on a layered construction^4^ of a Weyl semimetal, which enables the numerical simulation of Weyl semimetals with generic surface arcs. Using a recursive Greens function method, we numerically simulate the field dependence of the density of states in a magnetic field, and confirm the semiclassical predictions of the phase-space quantization scheme.

## Results

We first revisit the semiclassical quantization of cyclotron orbits, which generically demands that the phase difference between successive Landau levels is equal to 2*π*. The difference in phase accumulated between two successive levels for a fixed magnetic field can be expressed either in terms of the energy step and time or the difference in the product of momentum and displacement:


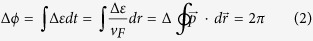


where 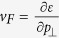 is the Fermi velocity, *p*_⊥_ is the momentum perpendicular to the orbit, and the last integral is over the spatial trajectory of the semiclassical orbit. For a simple derivation via path integral, see Supplementary information, Sec. I. Importantly, Eq. 2 is expressed through the difference in energy of neighboring Landau levels but makes no reference to their absolute position. While the overall energy scale is unimportant for, e.g. spectroscopy which probes only energy differences, quantum oscillations experiments are conducted by varying *B* at fixed chemical potential *μ*, such that the periodicity of quantum oscillations depends explicitly on the “zero of energy”.

Alternatively, the phase-space quantization framework offers an unambiguous reference to the energy, in which the momentum-displacement is integrated along the cyclotron orbit of constant energy contour at a specific chemical potential. In Supplementary information, we show how to reconcile these methods, however, in the mean time we proceed with the more transparent phase-space quantization approach.

**Figure 1:**
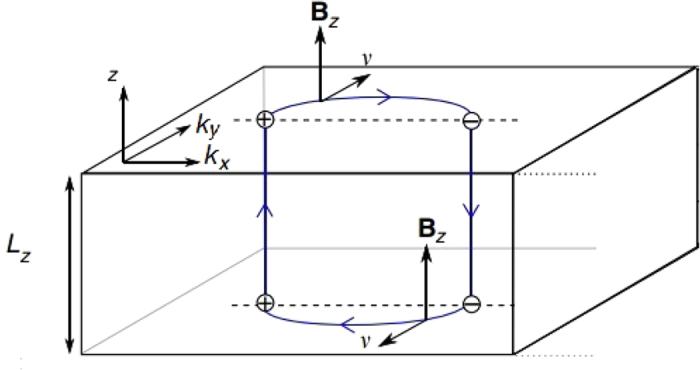
Schematic plot of a semiclassical orbit of a Weyl semimetal slab in a perpendicular magnetic field. The electrons traverse the Fermi arc on the top surface, travel through the one-dimensional chiral mode parallel to the magnetic field in the bulk, traverse the corresponding Fermi arc on the bottom surface, and then return along the opposite chiral mode through the bulk. Note that the real-space orbit in the *x* − *y* plane is rotated by 90°.

For the semiclassical cyclotron orbits described in ref. 9, the phase-space quantization condition is 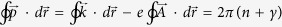, where the integral is over the four segments of the orbit: two Fermi arcs on the surfaces and two chiral modes in the bulk parallel to the magnetic field, as illustrated in Fig. 1. There may exist additional phase contributions at the turning points connecting the surface and bulk orbits, which are presumably constant for large enough *L*_*z*_ and can be absorbed into the constant *γ*. Throughout, we choose the convention that chemical potential, *μ*, is measured from the energy of the Weyl nodes in the bulk.

For the Fermi arcs, 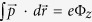, where Φ_*z*_ is the magnetic flux contained within the real-space orbit of area *S*_*R*_ in the *x* − *y* plane^12^. The semiclassical equations of motion imply


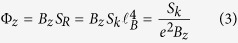


where *S*_*k*_ is the *k*-space area enclosed by the two Fermi arcs combined and *B*_*z*_ is the 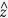 component of the magnetic field 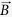. On the other hand, the chiral modes in the bulk are parallel to the magnetic field, so 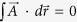 and:


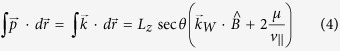


where *θ* is the tilting angle of 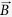 from the surface normal, 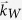 is the wave vector from + to − chirality Weyl nodes, and 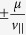 are the Fermi wave vectors of their respective chiral modes with velocity *v*_‖_ parallel to 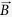, at chemical potential *μ*. Adding the contributions, phase-space quantization implies that quantum oscillations occur at fields:


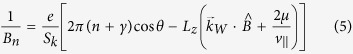


Eq. 5 is the main result of this paper.

## Discussion

### Comparison with and generalization to previous conclusions

When *μ* is close to the Weyl nodes and the Fermi velocity *v*_*s*_ is approximately constant along the surface Fermi arcs, we can expand 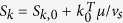, where *S*_*k*,0_ and 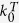 are the enclosed *k*-space area and total length of the combination of the two Fermi arcs from both surfaces for *μ* at the Weyl nodes. The frequency of the quantum oscillations 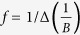 is


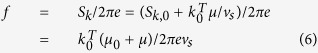


where 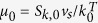. We see that our results reduce to those of ref. 9, under the special conditions: *S*_*k*_(*μ* = 0) = 0, 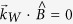, and *v*_*s*_ = *v*_‖_. However, the phase-space quantization method reveals two important generalizations:

1. The 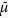 defined in Eq. 1 is generically not measured from the energy of the bulk Weyl nodes. In particular, if we require that *μ* is measured from the Weyl nodes, Eq. 1 should be modified by an offset 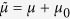. This reconciles the quantum oscillations from phase-space quantization and energy-time quantization: the contribution from the area *S*_*k*,0_ enclosed by the Fermi arcs at *μ* = 0 is reflected in *μ*_0_ while the contribution from the area change 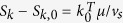 is reflected in *μ*. For cases where the area *S*_*k*,0_ is large in comparison with the area change, the inclusion of *μ*_0_ is necessary for the correct interpretation of the quantum oscillations.It is natural that *μ*_0_ should depend only on linearized Fermi-surface properties such as the area enclosed and the Fermi velocity, as the quantum oscillations generically encode only these low-energy universal features. We note that since *v*_*s*_ can in principle depend on chemical potential, *μ*, so does *μ*_0_ as defined above. For a quadratic surface dispersion, −*μ*_0_ can be interpreted as the energy (relative to the bulk Weyl nodes) at which the surface arcs enclose zero area perpendicular to the magnetic field. More generally, as we show in Supplementary information, the appropriate way to reconcile energy-time quantization is to set the zero of energy −*μ*_0_ at 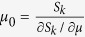, as the zero-area energy linearly extrapolated using the Fermi-surface property 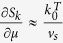.
2. The thickness of the Weyl semimetal slab *L*_*z*_ contributes to the quantum oscillations through the phase offset of 
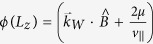
 *L*_*z*_ sec *θ*, which shifts the 1/*B* positions of the quantum oscillation peaks. Comparing to ref. 9, we see that the thickness dependent phase receives a contribution not only from the time 
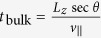
 taken to traverse the bulk via the chiral mode, but also from the momentum-space separation of the Weyl nodes projected onto 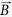. Interestingly, for fixed chemical potential *μ*, there exists a special cone of angles of 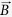, defined by: 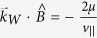, for which the phase vanishes, *ϕ*(*L*_*z*_) = 0, for all *L*_*z*_, such that the oscillations become independent of sample thickness.

### Experimental consequences

The new features revealed by the phase-space quantization treatment have several implications for experiments. As an example, the angle and thickness dependence of *ϕ*(*L*_*z*_), observable by tracking individual quantum oscillation peaks as a function of field orientation, can be used to quantitatively determine the *k*-space separation of the Weyl nodes from quantum oscillation measurements, which can be challenging to accurately extract from other probes such as photoemission.

#### Magic Angles

Moreover, the existence of a special set of angles for which the quantum oscillations become independent of sample thickness enables the following test: In recent experiments^10^, Moll *et al*. observed surface state oscillations in the Dirac semimetal Cd_3_As_2_ in thin film devices with parallel surfaces, which were absent in triangular devices with non-parallel top and bottom surfaces. The absence of oscillations in the latter triangular samples can be attributed to the destructive interference of orbits with different *L*_*z*_, due to the variation of device thickness along the triangle. The above computations predict that this geometric interference effect would be quenched for fields along the set of angles for which *ϕ*(*L*_*z*_) = 0, resulting in a reemergence of quantum oscillations. In contrast to the negative signature of not observing quantum oscillations in a triangular device, which could potentially arise from other extrinsic effects, such an observation would provide a clear positive signature of the non-local nature of the Weyl orbits. We note that observing this effect requires the field angle to be controlled to angular precision 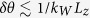, which may require thin devices.

#### Disorder and Thickness Dependence

As a final application, the phase-space quantization formulation above naturally reveals the effect of bulk disorder on dephasing the quantum oscillations associated with Weyl orbits. See Supplementary information, Sec. IV for a detailed discussion of disorder effects. For conventional magnetic orbits, any scattering from impurities strongly suppresses quantum oscillations, requiring 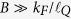 where 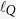 is the quantum mean-free path (distinct from the transport mean-free path, 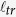, which only includes large-momentum transfer scattering) and *k*_*F*_ is the Fermi wave vector. As the surface-arc portion of the Weyl orbits is locally identical to conventional cyclotron motion, observing oscillations in the presence of disorder requires: 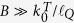. Naively, phase coherence along the bulk part similarly requires samples thinner than the quantum mean-free path, 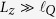, a potentially stringent condition since while typical Weyl materials have large 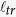, 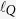 is typically much shorter^13^. However, the chiral nature of the bulk orbit along with the spatially correlated nature of disorder in low-density semimetals makes the bulk portion of the orbit more resilient to disorder effects.

Namely, for an electron traveling along a bulk chiral LL, a random potential *V* 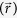, produces a local shift in the wave vector: 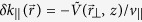, where 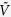 is the matrix element of the disorder potential in the chiral mode localized within 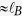 of transverse position 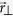 in the *xy*-plane. The total phase accumulated in this fashion is given by: 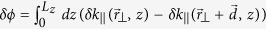, where the first term represents the random phase acquired traveling from bottom to top surface along the + chiral mode, and the second represents that of the return journey on the counter-propagating chiral mode of the opposite Weyl node. Between these two bulk legs of the orbit, the electron travels a spatial distance 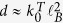 as it slides along the top surface Fermi arc. The typical disorder for low-density Weyl semimetals is poorly screened Coulomb impurities, which produce a potential that is spatially correlated over characteristic length scale 
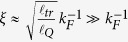
. For low-field, 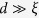, the two bulk legs of the orbit sample uncorrelated *V*, and dephasing indeed kills the quantum oscillations for 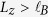. However, for higher fields 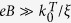, 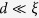 and the top-to-bottom and bottom-to-top legs accumulate nearly canceling random phases, which leads to the much weaker requirement on sample thickness, 
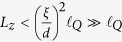
. For example, in Cd_3_As_2_, we estimate that the high-field regime is obtained for relatively low fields on the order of a few Tesla, in reasonable agreement with the observed field-scale at which surface-state oscillations onset in recent experiments^10^.

In conclusion, we have compared quantum oscillations with respect to the inverse magnetic field 1*/B* or chemical potential *μ*. The accurate definition of chemical potential and its reference point is vital for correctly converting between and reconciling different semi-classical quantization perspectives. For the quantum oscillations from the surface Fermi arcs and bulk chiral modes in Weyl semimetals, the general reference point of *μ* does not necessarily coincide with the Weyl points in the bulk. We derived the quantum oscillations using phase-space quantization conditions and proposed essential generalizations to previous conclusions and experimental consequences for generic Weyl semimetals. In the next section, we verify our claims numerically following the layered prescription.

## Methods

To verify the semiclassical predictions from the phase-space quantization approach we study a simple lattice model of Weyl semimetal following the layered prescription in ref. 4 and numerically calculate the density of states 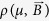 in a slab geometry. In the absence of the magnetic field, the Weyl semimetal is characterized by the following Hamiltonian:


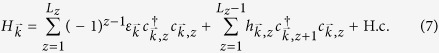


where 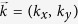 and the total number of layers *L*_*z*_ is odd so that the Fermi arcs on the top and bottom surfaces can be different^4^. We consider an in-plane dispersion 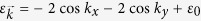 that represents nearest-neighbor hopping of amplitude −1 and an on-site energy of *ε*_0_. 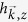 represents nearest-neighbor interlayer hopping with 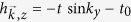 if *z* is odd and 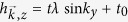 if *z* is even. We choose *λ* > 1, which ensures 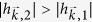 if *k*_*y*_ > 0 and vice versa. This model generates two Weyl nodes at 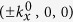 where 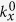 is the in-plane Fermi wave vector of 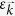 along the 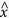 direction. By definition 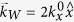. The surface Fermi arcs and the bulk chiral modes following the Weyl nodes are schematically consistent with the geometry in Fig. 1.

In the presence of a magnetic field 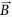, the translation symmetry in the 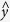 direction is preserved in the Landau gauge 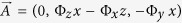, where Φ_*i*_ is the flux per plaquette perpendicular to the 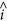 direction in units of the magnetic flux quantum, 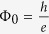, *i* = *x*, *y*, *z*. The Hamiltonian becomes:


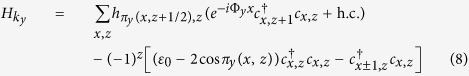


where *π*_*y*_(*x*, *z*) = *k*_*y*_ − *A*_*y*_(*x*, *z*).

The properties of this Hamiltonian such as the density of states 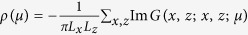 at the chemical potential *μ* can be calculated with the recursive Green’s function method where the real space degrees of freedom in the 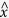 direction are treated recursively^14^,^15^,^16^. For an incommensurate flux Φ, physical properties of 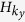 between different choices of *k*_*y*_ are equivalent in the thermodynamic limit^15^,^16^ and the summation over *k*_*y*_ can be neglected.

**Figure 2:**
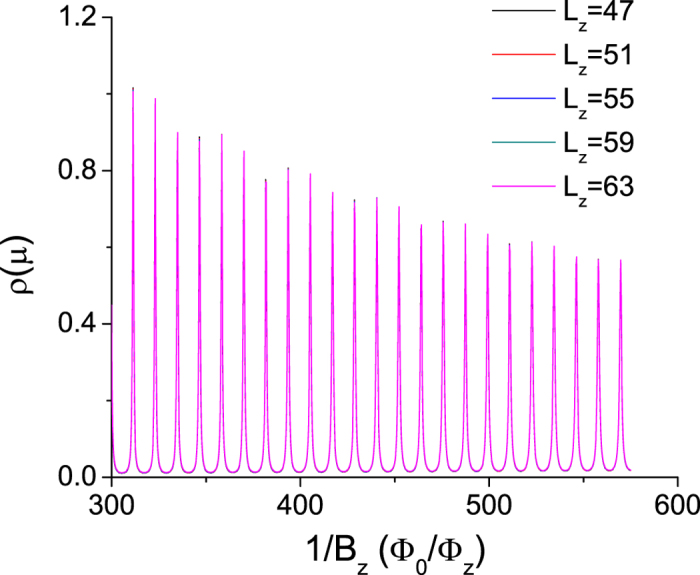
The density of states *ρ*(*μ*) versus the inverse magnetic field 1/*B*_*z*_ (in unit of Φ_0_/Φ_*z*_) for a Weyl semimetal slab of various thickness *L*_*z*_ shows clear quantum oscillations. The chemical potential *μ* = 0 is at the Weyl nodes. The characteristic quantum oscillation period is Δ(Φ_0_/Φ_*z*_) = 11.74.

We choose parameters *ε*_0_ = 3.0, *t* = 1.0, *t*_0_ = 2.0, *λ* = 2.0, and a small imaginary part *δ* = 0.001 in addition to the chemical potential *μ* as the level broadening. In this model, although the chemical potential is at the Weyl nodes, the Fermi arcs enclose a *k*-space area of 8.515% of the surface Brillouin zone. We first consider a magnetic field purely in the 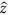 direction. Eq. 1 would predict no quantum oscillations if one assumed 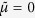, while Eq. 5 predicts quantum oscillations with a period Δ(Φ_0_/Φ_*z*_) = 11.74. The numerical results of the density of states *ρ*(*μ*) versus the inverse magnetic field 1/*B* and various slab thickness *L*_*z*_, shown in Fig. 2, show clear signatures of quantum oscillations whose period is in quantitative agreement with our formula.

**Figure 3:**
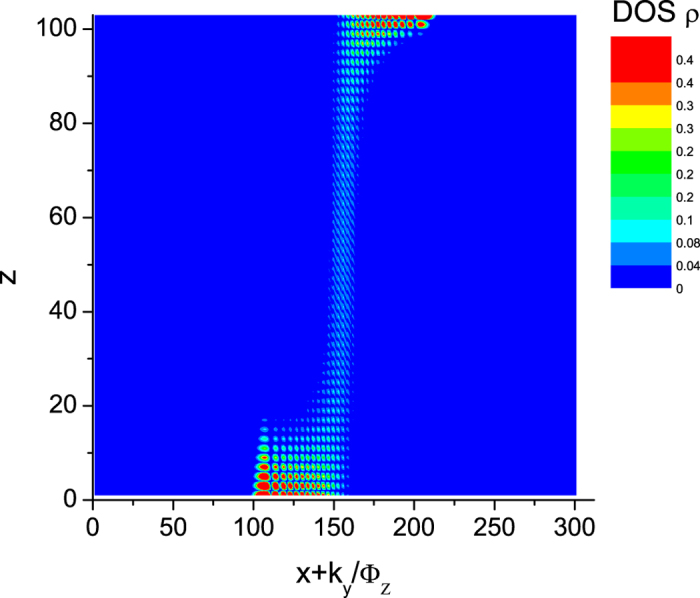
The local density of states distribution in the *x* − *z* plane at Φ_0_/Φ_*z*_ = 311.40, *μ* = 0 and *L*_*z*_ = 103 is consistent with the cyclotron orbit illustrated in Fig. 1 and clearly consists of components from the Fermi arcs on both Fermi surfaces and chiral LL modes in the bulk.

To verify that the semiclassical orbit contains components in the bulk as well as on both of the top and bottom surfaces, we calculate the local density of states distribution 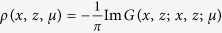 in the *x* − *z* plane with the *x* coordinate replaced by *x* + *k*_*y*_/Φ_*z*_. The result for Φ_0_/Φ_*z*_ = 311.40, *μ* = 0 and *L*_*z*_ = 103 is shown in Fig. 3. The Fermi arcs are at *k*_*y*_ > 0 and *k*_*y*_ < 0 for the top and bottom surfaces as well as the chiral modes in the bulk are clearly visible.

**Figure 4:**
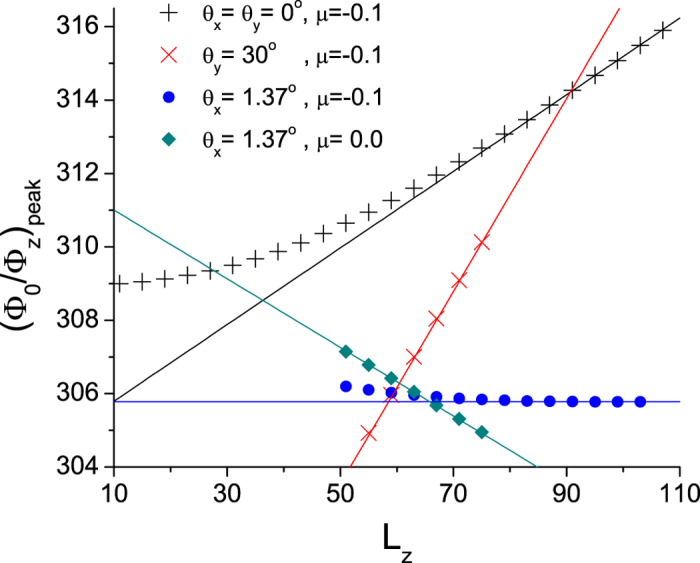
Symbols mark the location of one of the density of states peaks as a function of the slab thickness *L*_*z*_ at different chemical potential *μ* and magnetic field tilting angle *θ*_*y*_ or *θ*_*x*_. The lines are the asymptotic expression in the large *L*_*z*_ limit derived from the positions of and the Fermi velocity around the Weyl nodes in the bulk: 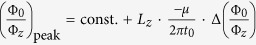 for a magnetic field in the 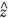 direction, and refer to Supplementary information for the expressions in the presence of a tilted magnetic field. While the peak positions typically show strong *L*_*z*_ dependence, notably, for particular ‘magic’ angles (blue circles), the peak positions asymptotically become nearly independent of sample thickness.

In addition, Eq. 5 suggests that the thickness of the slab *L*_*z*_ changes the phase of the quantum oscillations, and thus the actual locations of the *ρ*(*μ*) peaks in Φ_0_/Φ_*z*_. For a magnetic field in the 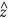 direction and *μ* = 0, 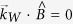, we expect no *L*_*z*_ dependence, which is confirmed in Fig. 2. For a finite *μ* and a field in the 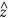 direction, however, the shift *δ*(1/*B*) of the peak positions is given by


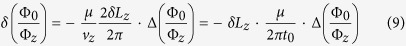


where 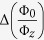 is the period of the quantum oscillations. We numerically observe this shift in the locations of the quantum oscillation peaks at *μ* = −0.1 in Fig. 4, where the location of one of the peaks is tracked as *L*_*z*_ is varied. The deviation from Eq. 9 at small *L*_*z*_ is due to the finite extent of the edge states (Fig. 3). At relatively large *L*_*z*_ where the physics in the center of the slab can be approximately treated as in the bulk, Eq. 9 gives an accurate description of the *L*_*z*_ dependence of the quantum oscillation phenomena. The above conclusions also hold true for a magnetic field that is tilted in the 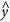 direction, e.g. 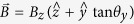, where only the *L*_*z*_ coefficient is modified, see Fig. 4.

In comparison, the magnetic field tilted in the 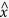 direction gives qualitatively different behavior, since 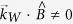 along 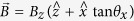. First, there exists *L*_*z*_ dependence 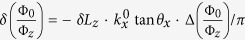 for chemical potential *μ* = 0 at the energy of the Weyl nodes. Interestingly, for a given chemical potential *μ*, such *L*_*z*_ dependence vanishes at a special tilting angle 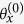, which satisfies


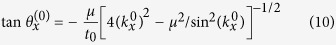


Numerical results for *μ* = 0 and *μ* = −0.1 confirm these expectations at tilting angle 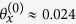 as in Eq. 10 for *μ* = −0.1 (Fig. 4). Derivations and further discussion on the *L*_*z*_ dependence of the peak positions in a tilted magnetic field 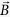 are in Supplementary information.

## Additional Information

**How to cite this article**: Zhang, Y. *et al*. Quantum oscillations from generic surface Fermi arcs and bulk chiral modes in Weyl semimetals. *Sci. Rep.* **6**, 23741; doi: 10.1038/srep23741 (2016).

## Supplementary Material

Supplementary Information
