# Supplementary material for: Quantum oscillations from generic surface Fermi arcs and bulk chiral modes in Weyl semimetals
Source: Sci Rep. 2016 Apr 1;6:23741. doi: 10.1038/srep23741 (PMC4817138; doi:10.1038/srep23741)
Supplement: Supplementary Information [file srep23741-s1.pdf]

Supplementary information: Quantum oscillations from generic surface Fermi arcs and bulk chiral modes in Weyl semimetals

Yi Zhang[1], Daniel Bulmash[1], Pavan Hosur[1], Andrew C. Potter[2], and Ashvin Vishwanath[2]
[1] *Department of Physics, Stanford University, Stanford, California 94305, USA and*
[2] *Department of Physics, University of California, Berkeley, California 94720, USA.*
(Dated: February 8, 2016)

I. PATH INTEGRAL DERIVATION OF ENERGY-TIME QUANTIZATION

Suppose that we have a quantum system with Hamiltonian H and corresponding classical action S. Let the eigenstates of H be $|E\rangle$. Then the propagator $U(x, x'; t)$ obeys

$$U(x, x; T) = \sum_{E} |\langle x|E\rangle|^2 e^{-iET} \tag{1}$$

Computing the propagator in the path integral instead, we can make a semiclassical approximation and assume that the only paths which contribute are those paths $x_{cl}(t)$ which start at x and return there in time T according to the classical equations of motion. In this approximation, we get an alternative expression for the same function:

$$U(x, x; T) \approx \mathcal{N} \sum_{x_{cl}(t)} e^{iS[x_{cl}(t)]} \tag{2}$$

where $\mathcal{N}$ is a prefactor irrelevant to us.

Now come the main assumptions. Inspired by the free particle in a magnetic field, for which these assumptions definitely hold, assume that there are no nonstationary closed classical paths except when $T = nT_0$ where T_0 is some classical period. Furthermore, we assume that each classical path at $T = nT_0$ is just n loops of a single closed orbit $x_\alpha(t)$ which traverses the loop once in time T_0. In this case, absorbing any prefactors into $\mathcal{N}$,

$$U(x, x; T) \approx \mathcal{N} \sum_{\alpha} \sum_{n} e^{inS[x_\alpha(t)]} \delta(T - nT_0) \tag{3}$$

$$= \mathcal{N} \sum_{\alpha} \int d\omega e^{-i\omega T} \sum_{n} e^{in(\omega T_0 + S[x_\alpha])} \tag{4}$$

$$= \mathcal{N} \sum_{\alpha,m} \int d\omega e^{-i\omega T} \delta(\omega T_0 + S[x_\alpha] - 2\pi m) \tag{5}$$

Comparing to the exact propagator Eq. 1, we see then that energies are labeled by m and α and are given by

$$E_{m,\alpha} = \frac{2\pi m - S[x_\alpha]}{T_0} \tag{6}$$

This implies the energy-time quantization condition in Eq. 2 in the main text, but also tells us more. Here $S[x_\alpha]$ explicitly depends on the in-field Hamiltonian and thus the zero of energy for the Landau levels, so $E_{m,\alpha}$ is automatically defined relative to the same zero. In particular, if we assume $T_0 \sim 1/B$, then in deriving a condition like Eq. 1 in the main text the energy μ must be defined relative to the Landau level spectrum, which need not coincide with the natural zero of energy for the $B = 0$ band structure.

II. QUANTUM OSCILLATIONS FROM ENERGY-TIME QUANTIZATION

In this section, we compare the quantum oscillations from the two different perspectives of energy-time quantization and phase-space quantization. The phase-space quantization gives the condition of the allowed semiclassical orbits:

$$\oint (\vec{k} - e\vec{A}) \cdot \mathrm{d}\vec{r} = 2\pi(n + \gamma) \tag{7}$$

where the integral is over a constant energy contour in k-space at certain chemical potential μ. With the semiclassical equations of motion, this can be re-written as:

$$\ell_B^2 S_k = \frac{\ell_B^2}{2} \oint k_\perp \mathrm{d}k_\| = 2\pi(n+\gamma) \tag{8}$$

where $k_\perp$ and $k_\|$ are the wave vectors normal and parallel to the constant energy contour, respectively, S_k is the enclosed k-space area and $\ell_B = 1/\sqrt{eB}$ is the magnetic length.

On the other hand, consider a dispersion $\varepsilon(\vec{k})$ in two dimensions, the time needed to complete a cyclotron orbit at energy ε_n is

$$t = \ell_B^2 \oint \frac{\mathrm{d}k_\|}{v_\perp(\vec{k})} \tag{9}$$

where $v_\perp$ is the Fermi velocity perpendicular to the contour. The energy-time quantization states that $(\varepsilon_n - \mu_0)\, t = 2\pi(n+\gamma)$, which suggests:

$$\ell_B^2 \oint \frac{\varepsilon_n - \mu_0}{v_\perp(\vec{k})} \mathrm{d}k_\| = 2\pi(n+\gamma) \tag{10}$$

where μ_0 is a constant offset and γ is a Berry phase contribution.

In particular, the two conditions in Eq. 8 and 10 match when

$$\frac{\varepsilon_n - \mu_0}{v_\perp} = \frac{k_\perp}{2} \tag{11}$$

For a parabolic dispersion, e.g., a two-dimensional electron gas $\varepsilon_{\vec{k}} = k^2/2m$, this is consistent with $\mu_0 = 0$. Namely, the zero of energy is at the bottom of the band.

For a more generic dispersion, we assume the Fermi velocity $v_\perp$ is independent of ε_n in a small range around the chemical potential ε_F, then it is straightforward to take a derivative with respect to n and get:

$$\mu_0 \frac{\mathrm{d}}{\mathrm{d}n} \oint \frac{\mathrm{d}k_\|}{v_\perp(\vec{k})} = \frac{\mathrm{d}}{2\mathrm{d}n} \oint \frac{\mathrm{d}k_\|}{v_\perp(\vec{k})} = \frac{\mathrm{d}S_k}{\mathrm{d}n}$$

$$\mu_0 = S_k / \oint \frac{\mathrm{d}k_\|}{v_\perp(\vec{k})} = S_k \cdot \frac{\mathrm{d}\mu}{\mathrm{d}S_k} \tag{12}$$

Physically, given the Fermi surface area S_k and its derivative $\frac{\mathrm{d}S_k}{\mathrm{d}\mu}$ with respect to the chemical potential μ near the Fermi level, the linear extrapolation to lower energies gives the zero of energy as where the cross-section area of the constant energy contour vanishes. For a linear dispersion $\varepsilon = \pm v \left|\vec{k}\right|$ at chemical potential $\varepsilon_F = v k_F$, for example, the zero of energy is not at the Dirac node. It is straightforward to show that the consistent quantum oscillations are derived from energy-time quantization with $\mu_0 = \varepsilon_F/2 = v k_F/2$.

Therefore, it is vital to understand where is the zero of energy that the chemical potential μ is measured from. As an example of the importance and ambiguity in correctly defining the zero of energy, the quantum oscillations of a two-dimensional electron gas $\varepsilon_{\vec{k}} = k^2/2m$ is:

$$\frac{1}{B_n} = \frac{2\pi e}{S_k}\left(n + \frac{1}{2}\right) = \frac{2e}{k_F^2}\left(n + \frac{1}{2}\right) \tag{13}$$

where $S_k = \pi k_F^2$ and k_F is the Fermi wave vector.

The energy-time quantization leads to the Landau levels:

$$\varepsilon_n = \omega_c (n+\gamma) - \mu_0 \tag{14}$$

where $\omega_c = \frac{eB}{m}$ is the cyclotron frequency, and μ_0 and γ are unknown constants since $\Delta\varepsilon \times t = 2\pi$ only gives the quantized level spacings $\Delta\varepsilon = \varepsilon_n - \varepsilon_{n-1}$ and contains no information on the exact zero of energy. For the quantum oscillations at a fixed chemical potential $\mu = k_F^2/2m$, set $\varepsilon_n = \mu$:

$$\begin{aligned} \frac{k_F^2}{2m} &= \frac{eB_n}{m}(n+\gamma) - \mu_0 \\ \frac{1}{B_n} &= \frac{2e}{k_F^2 + 2m\mu_0}(n+\gamma) \end{aligned} \tag{15}$$

identical to Eq. 13 if we set $\gamma = 1/2$ and $\mu_0 = 0$ as we have derived above. Importantly, the fundamental behaviors of the quantum oscillations including its characteristic frequency are not consistently recovered by this formula if $\mu_0 \neq 0$.

III. THICKNESS DEPENDENCE IN A TILTED MAGNETIC FIELD

We can linearize the dispersion near the Weyl nodes for the lattice model we consider in the main text:

$$\varepsilon_{\vec{k}}^{\pm}=\left[\left(2t_0k_z\right)^2+\left(\left(\lambda-1\right)tk_y\right)^2+\left(\pm 2\sin k_x^0k_x\right)^2\right]^{1/2} \tag{16}$$

where $k_x^0=\cos^{-1}\left(\varepsilon_0/2-1\right)$ and the $\pm$ signs are for the two Weyl nodes with opposite chirality.

For a magnetic field tilted in the $\hat{y}$ direction $\vec{B}=B_z\left(\hat{z}+\hat{y}\tan\theta_y\right)$, the Fermi wave vector of the chiral modes are

$$\vec{k}_{\parallel,1(2)}=\pm\frac{\mu}{2\left(\lambda-1\right)tt_0}\frac{4t_0^2\tan\theta_y\hat{y}+\left(\lambda-1\right)^2t^2\hat{z}}{\left[4t_0^2\tan^2\theta_y+\left(\lambda-1\right)^2t^2\right]^{1/2}} \tag{17}$$

The shift of the peak positions is

$$\delta\left(\frac{\Phi_0}{\Phi_z}\right)=-\frac{\mu\cdot\delta L_z}{2\pi\left(\lambda-1\right)tt_0}\left[4t_0^2\tan^2\theta_y+\left(\lambda-1\right)^2t^2\right]^{1/2}\Delta\left(\frac{\Phi_0}{\Phi_z}\right) \tag{18}$$

Similarly, for a magnetic field tilted in the $\hat{x}$ direction $\vec{B}=B_z\left(\hat{z}+\hat{x}\tan\theta_x\right)$, the Fermi wave vector of the chiral modes are

$$\vec{k}_{\parallel,1(2)}=\pm\frac{\mu}{2t_0\sin k_x^0}\frac{t_0^2\tan\theta_x\hat{x}+\sin^2k_x^0\hat{z}}{\left[t_0^2\tan^2\theta_x+\sin^2k_x^0t^2\right]^{1/2}} \tag{19}$$

together with the location of the Weyl nodes at $\left(\pm k_x^0,0,0\right)$ therefore $\vec{k}_W=2k_x^0\hat{x}$, the shift of the peak positions is

$$\delta\left(\frac{\Phi_0}{\Phi_z}\right)=-\left[\frac{\mu}{2t_0\sin k_x^0}\left(t_0^2\tan^2\theta_x+\sin^2k_x^0\right)^{1/2}+k_x^0\tan\theta_x\right]\frac{2\delta L_z}{2\pi}\cdot\Delta\left(\frac{\Phi_0}{\Phi_z}\right)$$

There exists a residual L_z dependence $\delta\left(\frac{\Phi_0}{\Phi_z}\right)=-\delta L_z\cdot k_x^0\tan\theta_x\cdot\Delta\left(\frac{\Phi_0}{\Phi_z}\right)/\pi$ at $\mu=0$. In addition, at tilting angle $\theta_x^{(0)}$ satisfying

$$\tan\theta_x^{(0)}=-\frac{\mu}{t_0}\left[4\left(k_x^0\right)^2-\mu^2/\sin^2\left(k_x^0\right)\right]^{-1/2} \tag{20}$$

the coefficient in the square bracket vanishes, and the quantum oscillations have no manifest L_z dependence.

IV. DISORDER EFFECTS

A. Disorder Model

As a model for the bulk effects of disorder, consider Weyl nodes with linear dispersion, and a random potential $V(r)$ with Gaussian distribution characterized by correlations $\overline{V(r)V(r')}=V_0^2f^{(3)}(r-r')$, where $f^{(d)}(r)$ is a smooth function that decays with characteristic length scale ξ, which, for concreteness, we will take as a normalized d-dimensional Gaussian with variance ξ^2, and $\overline{(\cdots)}$ indicates an average over disorder configurations.

In the Born approximation, the quantum lifetime, τ_Q, characterizing the timescale between elastic scattering events in the bulk in the absence of a field is:

$$\tau_Q^{-1}=\frac{2\pi\nu(0)V_0^2}{(k_F\xi)^2} \tag{21}$$

which is related to the quantum mean-free path by $\ell_Q=\frac{\tau_Q}{v}$, where v_F is the geometric average of the different spatial components of the bulk velocity. Here $\nu(0)=\frac{k_F^2}{2\pi^2v_F}$ is the density of states for the Fermi-surface of a single Weyl node, and $k_F=\frac{\mu}{v_F}$ is the Fermi wave vector for the Weyl pocket.

In contrast, the transport lifetime obtained by weighting scattering events between states with momenta $\vec{k}$ and $\vec{k}'$ by a factor of $\left(1-\cos\theta_{\vec{k},\vec{k}'}\right) \approx \left(\frac{1}{k_F\xi}\right)^2$, where $\theta_{\vec{k},\vec{k}'}$ is the angle between $\vec{k}, \vec{k}'$, is given by:

$$\tau_{\text{tr}}^{-1} = \frac{1}{(k_F\xi)^2}\tau_Q^{-1} \tag{22}$$

We can determine the parameter V_0^2 in terms of the measurable quantities τ_Q^{-1}:

$$V_0^2 = \frac{\pi v_F \xi^2}{\tau_Q} \tag{23}$$

B. Different Scattering Processes

For the bulk portion of the Weyl orbit, there are three potentially detrimental sources of disorder induced dephasing: 1) intervalley scattering between opposite chirality Weyl nodes, 2) scattering between different Landau levels (LLs) within a single Weyl node, and 3) random phase accumulated along the chiral LLs in the absence of inter-LL scattering. We will consider each of these channels in turn. The rates for these dephasing channels add, indicating that their inverse length scales add: $\ell_{\text{tot}} = \left(\sum_i \frac{1}{\ell_i}\right)^{-1}$, indicating that the sample thickness limitation on observing quantum oscillations will be set by the shortest scattering length scale.

C. Intervalley Scattering

As intervalley scattering requires momentum transfer $\approx k_W$, for long-wavelength disorder, intervalley scattering will be suppressed by $\frac{1}{(k_W\xi)^2}$ compared to total quantum scattering, indicating:

$$\ell_{\text{inter-valley}} \approx (k_W\xi)^2\,\ell_Q \tag{24}$$

In particular, since $k_W > k_F$, this length scale is even longer than the transport mean free path, $\ell_{\text{tr}} \approx (k_F\xi)^2\ell_Q$, for all field strengths.

D. Inter Landau level Scattering

For μ larger than the LL spacing, multiple non-chiral bulk LLs will coexist at the same energy as the chiral modes, and scattering between chiral and non-chiral modes within the same Weyl node is possible. However, since the wavefunctions of different LL modes differ on lengthscales of order ℓ_B, for $\xi \gg \ell_B$, the matrix element for inter-LL scattering between levels with indices m and n is suppressed by approximately a factor of $\left(\frac{\ell_B}{\xi}\right)^{|m-n|}$:

$$\begin{aligned} V_{n,m} &= \langle u_n|V|u_m\rangle \\ &\approx \int dx \frac{e^{-x^2/2\ell_B^2}}{2\pi\ell_B^2}\sum_r H_n(x)H_m(x)\frac{x^r V^{(r)}(0)}{r!} \\ &\approx \left(\frac{\ell_B}{\xi}\right)^{|m-n|} V_0 \end{aligned} \tag{25}$$

where $H_n(x)$ is the nth Hermite polynomial. The dominant mixing will hence come from minimal difference in LL indices, and hence, for $\ell_B \ll \xi$, the inter-LL scattering rate is suppressed by a factor of $\frac{|V_{0,1}|^2}{|V_0|^2} \approx \left(\frac{\ell_B}{\xi}\right)^2$, i.e. we expect:

$$\ell_{\text{inter-LL}} \approx \left(\frac{\xi}{\ell_B}\right)^2 \ell_Q \tag{26}$$

We will see below that this length scale is longer than that set by dephasing while propagating along the chiral bulk modes, which is expected to be the dominant limiting factor in observing quantum oscillations.

E. Dephasing within the bulk chiral modes

In the previous section, we have seen that inter-LL scattering may be neglected for $\ell_B \ll \xi$, and $L_z \ll \ell_{\text{inter-LL}}$. In this regime, the bulk portion of the orbit occurs purely within the chiral modes of the lowest Landau level. In the presence of an impurity potential $V(\vec{r})$ that varies smoothly on the length scale of k_F^{-1}, we can model the $\pm$ chiral mode localized within ℓ_B of the guiding center position $\vec{r}_\perp$ in the xy plane, by the continuum Hamiltonian:

$$H_{ch}^{(\pm)}(\vec{r}_\perp) = \psi^\dagger \left(\mp i v_F \partial_z - U(\vec{r}_\perp, z)\right)\psi \tag{27}$$

where $U(\vec{r}_\perp, z) = \int d^2\delta r_\perp V(r_0 + \delta r_\perp, z)|u_0(r_\perp)|^2$ is the matrix element of V within the lowest LL orbital with guiding center coordinate $\vec{r}_\perp$ which has wave function $u_0(r_0 + \delta r_\perp) \sim \frac{1}{4\pi\ell_B^2} e^{-\delta r_\perp^2/(4\ell_B^2)}$.

For moderate fields, $\ell_B \ll \xi$, the mean-square of these matrix elements is then given by:

$$\overline{U(r,z)U(r,z')} \approx \frac{V_0^2}{2\pi\xi^2} f^{(1)}(z-z') \tag{28}$$

The random phase factor accumulated through the bulk portion of the orbit (ignoring mixing between chiral and non-chiral levels) is:

$$e^{i\delta\phi} = \exp\left[i\int_0^L \frac{U_{r_\perp}(z) - U_{r_\perp + d}(z)}{v_F}\right] \tag{29}$$

where the first term comes from propagating from bottom to top surface along the + chiral LL, and the second comes from returning from top to bottom surface along the counter-propagating $-$ chiral LL. In between the electron travels spatial distance $d = k_0^T \ell_B^2$ as it slides along the surface arc of the top surface.

Averaging the disorder phase over disorder gives:

$$\overline{e^{i\delta\phi}} = e^{-\frac{1}{2v_F^2}\int_0^{L_z} dz dz' \overline{(U(r,z)-U(r+d,z))\left(U(r,z')-U(r+k_0\ell_B^2,z')\right)}} \tag{30}$$

The suppression factor depends strongly on the ratio of the orbit size, $d = k_0^T \ell_B^2$, to the disorder correlation length.

1. *Low field regime* ($d \gg \xi$)

In the low field regime, $d \gg \xi$, $U(r,z)$ is uncorrelated with $U(r + k_0\ell_B^2, z)$, and:

$$\overline{e^{i\delta\phi}} \approx e^{-\frac{2}{v_F^2}\int_0^L dz dz' \overline{U(r,z)U(r,z')}} \approx e^{-\frac{v_F^2 V_0^2}{\pi\xi^2} L_z} \tag{31}$$

from which we identify the relevant bulk "mean-free path" length scale:

$$\ell_{ch} = \frac{\pi v_F^2 \xi^2}{V_0^2} = v_F \tau_Q = \ell_Q \tag{32}$$

which is just the quantum mean-free path.

2. *High field regime* ($d \ll \xi$)

On the other hand, for strong fields, $d \ll \xi$, the phase accumulated in traversing from bottom to top surfaces samples almost the same disorder configuration as the reverse trip, resulting in near cancellation of the total accumulated phase, and leading to a longer effective dephasing length for the chiral channel $\ell_{ch} \gg \ell_Q$.

To estimate ℓ_{ch}, in this regime we also need the expression for:

$$\overline{U(r,z)U(r+d,z')} \approx \left[1 - \left(\frac{d}{\xi}\right)^2\right] \overline{U(r,z)U(r,z')} \tag{33}$$

with which we find:

$$\overline{e^{i\delta\phi}} \approx e^{-\frac{1}{v_F^2}\int_0^{L_z} dzdz' \left(\overline{U(r,z)U(r,z')-U(r,z)U(r+k_0\ell_B^2,z')}\right)}$$
$$\approx e^{-\left(\frac{d}{\xi}\right)^2 \frac{L_z}{\ell_Q}} \equiv e^{-L_z/\ell_{ch}} \quad (34)$$

Hence, in the high-field regime, the chiral nature of the bulk LLs and long correlation length of disorder enables quantum oscillations to be observed for sample thicknesses up to

$$\ell_{ch} \approx \left(\frac{\xi}{d}\right)^2 \ell_Q \quad (35)$$

which can substantially exceed the quantum mean-free path.

We can express the enhancement of the dephasing length for the bulk chiral modes, ℓ_{ch}, compared to the quantum mean-free path, ℓ_Q, in terms of measurable quantities including: 1) the ratio $\left(\frac{\tau_{tr}}{\tau_Q}\right)$ of transport to quantum lifetimes obtained respectively from transport and bulk quantum oscillation measurements, and 2) the frequency of the surface oscillations $f \approx k_F k_0$, as:

$$\frac{\ell_{ch}}{\ell_Q} = \left(\frac{\xi}{d}\right)^2 \approx \frac{\sqrt{\tau_{\rm tr}/\tau_Q}}{f/B} \quad (36)$$

We note that the "high-field" regime may be accessed for relatively modest field scales. For example, for Cd_3As_2, $k_F\xi \approx \sqrt{\frac{\tau_{\rm tr}}{\tau_Q}} \approx 10-30$, [1] and $f \approx 60T$, [2] and we estimate that $d \gg \xi$ can be achieved for fields of order a few Tesla, which is incidentally consistent with the lowest fields for which surface oscillations are seen in recent thin film devices[2].

[1] Tian Liang, et al. Ultrahigh mobility and giant magnetoresistance in the Dirac semimetal Cd3As2. Nature Materials 14, 280-284 (2015).
[2] Philip J. W. Moll, et al. Chirality transfer dynamics in quantum orbits in the Dirac semi-metal Cd3As2. eprint-arXiv:1505.02817 (2015).
